# The Occurrence of Propyl Lactate in Chinese Baijius (Chinese Liquors) Detected by Direct Injection Coupled with Gas Chromatography-Mass Spectrometry

**DOI:** 10.3390/molecules201019002

**Published:** 2015-10-19

**Authors:** Jihong Wu, Yang Zheng, Baoguo Sun, Xiaotao Sun, Jiyuan Sun, Fuping Zheng, Mingquan Huang

**Affiliations:** 1 School of Food and Chemical Engineering, Beijing Technology and Business University, Beijing 100048, China; E-Mails: wujihong12@126.com (J.W.); an2zhengyang@126.com (Y.Z.); sunbg@btbu.edu.cn (B.S.); sunxiaotao@th.btbu.edu.cn (X.S.); sunjinyuan@th.btbu.edu.cn (J.S.); zhengfp@th.btbu.edu.cn (F.Z.); 2 Beijing Key Laboratory of Flavor Chemistry, Beijing Technology and Business University, Beijing 100048, China; 3 Beijing Innovation Centre of Food Nutrition and Human Health, Beijing 100048, China

**Keywords:** Chinese Baijiu, propyl lactate, gas chromatography-mass spectrometry (GC-MS), threshold, odor activity values (OAVs)

## Abstract

As one of the oldest distillates in the world, flavor compounds of Chinese Baijiu (Chinese liquor) were extremely complex. Propyl lactate was firstly detected by direct injection and gas chromatography-mass spectrometry (GC-MS) in 72 Chinese Baijius. The objectives were to detect the contents of propyl lactate and evaluate its contribution to the aroma of Chinese Baijiu based on odor activity values (OAVs). The levels of propyl lactate in these distillates were determined by internal standard method and selective ion monitoring (SIM), which ranged from 0.050 to 1.900 mg∙L^−1^ under investigation. Its detection threshold was determined by Three-Alternative Forced-Choice (3-AFC) and curve fitting (CF), which was 0.740 mg∙L^−1^ in 38% ethanol solution. The contribution of propyl lactate on the aroma of these distillate drinks was evaluated by their odor activity values (OAVs), which varied from 0.066 to 4.440. The OAVs of propyl lactate were found to exceed 1 in 13 Chinese Baijius, including 50° Jingzhi Guniang 5 years (4.440), 52° Jingzhi Guniang 10 years (3.024), Jingyanggang (2.568), Xianghe Ronghe Shaofang (2.313), and 1956 Laolang (1.431), which indicated that propyl lactate was one of odor-active components in these Chinese Baijius.

## 1. Introduction

Chinese Baijiu (Chinese liquor) is one of the oldest distillates in the world, and it is the most popular spirits in China with the annual production of about 4 million metric tons. The general process for the production of Chinese Baijiu is as follows. At first, the grain raw materials, such as wheat, sorghum, corn, rice or glutinous rice, are cooked with steam. And then some saccharification and fermentation agents (“DaQu” or “XiaoQu”) are added into the cooked grain matrix. Finally, the liquors are distilled out with steam from the fermentation products after several months or years of fermentation [1]. The fresh distillates need to be aged for a long time in order to balance the flavors. The final commercialized products are blended with aged distillate drinks, fresh distillate drinks and water based on certain ratios according to different formulations of spirit drinks [2].

Flavor compounds of Chinese Baijiu are extremely complex due to different raw materials, various microorganisms and diverse procedures in different production regions, and a great number of compounds have been studied extensively [3,4,5,6], such as esters, alcohols, ketones, acids, and so on. Lactates have been reported to occur in Chinese liquors widely, such as methyl lactate [4], ethyl lactate [7], butyl lactate [8], hexyl lactate [9], isopropyl lactate [10], isobutyl lactate [4] and isoamyl lactate [4]. Lactates are recognized to be important flavor compounds in Chinese Baijiu [11]. Ethyl lactate and butyl lactate were detected in “Gujing” Baijiu during investigation carried out in our lab. In the meantime, lactic acid and propanol were also found in this distillate, which had been reported in other Chinese Baijius [12,13]. It is a little strange that propyl lactate has not been reported in Chinese Baijiu up to now. The same are other alcoholic beverages, except distilled Calvados [14] and Chinese rice wine [15]. Meanwhile, the odor properties of propyl lactate and its contribution to aroma of these spirits are also not known. We checked the newest edition (2011 edition) of NIST (National Institute of Standards and Technology) library and found that the mass spectrogram of propyl lactate was not included in this library. The occurrence of propyl lactate in Chinese Baijiu might be overlooked since the identification of volatile components was usually performed by searching NIST library. The objective of this work were (i) the detection of the occurrence of propyl lactate in 72 Chinese Baijius and (ii) the evaluation of its contribution to the aroma of Chinese Baijius based on odor activity values (OAVs).

## 2. Results and Discussion

### 2.1. Identification of Propyl Lactate in Distillate Samples

**Figure 1:**
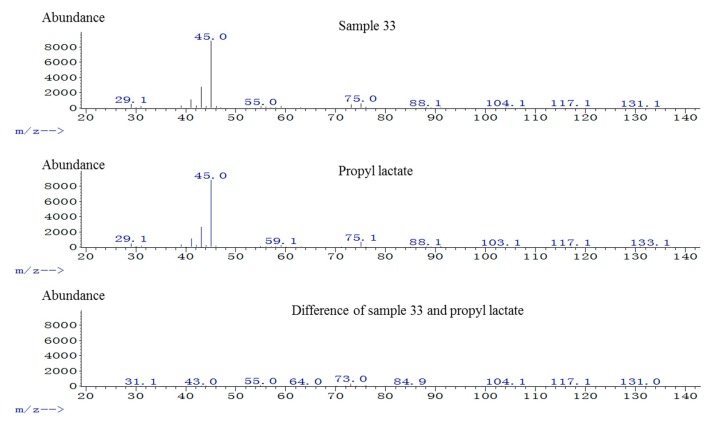
TIC of propyl lactate in GC-MS. The mass spectra of an unknown compound shown at 17.4 min in sample 33 and propyl lactate standard are presented. The differential spectrum of them is located at the bottom.

**Figure 2:**
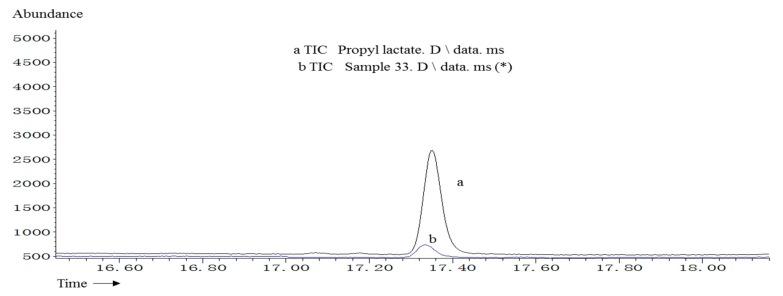
The magnifying TIC of sample 33 and propyl lactate at 17.4 min. The two magnified TICs are shown, one is the unknown compound in sample 33 (a TIC), and the other is propyl lactate standard (b TIC) at 17.4 min.

The mass spectra of an unknown compound shown at 17.4 min in sample 33 and propyl lactate standard were presented as Figure 1, and the differential spectrum of them was located at the bottom, which indicated the unknown compound shown at 17.4 min being matched with propyl lactate. The two magnified TICs of the unknown compound in sample 33 (a TIC) and propyl lactate standard (b TIC) at 17.4 min were shown in Figure 2. These two peaks were completely overlapped.

### 2.2. Quantitative Analysis of Propyl Lactate in Distillate Samples

The concentrations of propyl lactate were determined in these spirit drinks by the internal standard method with the selected ions monitoring mode of GC-MS. The LOD and the LOQ of the method were 0.025 mg∙L^−1^ and 0.050 mg∙L^−1^, respectively. The internal standard curve equations included three equations in different concentration ranges, including *y* = 4.4587*x* + 0.0112 (0.050 < *y* < 0.60), *y* = 1.9987*x +* 0.2673 (0.60 < *y* < 1.00) and *y* = 2.1972*x* + 0.0569 (1.00 < *y* < 4.50). The corresponding correlation coefficients (R^2^) were 0.9994, 0.9917 and 0.9993, respectively. The quantitative results of propyl lactate were shown in Table 1. The content of propyl lactate in other 10 Chinese Baijiu, numbered 73 to 82, were also included in Table 1, which our lab had reported before [16].

**Table 1:** The concentrations of propyl lactate in 82 Chinese Baijiu samples (Detection threshold 0.740 mg∙L^−1^).

No.	Sample Name (Alcohol % by Volume)	Manufacturer	Concentrations of Propyl Lactate (mg∙L^−1^)	OAV of Propyl Lactate
1	Guizhou Yuanjiang Chenniang 18 Years (52°)	Kweichow Moutai Co., Ltd.	0.064 ± 0.003	0.086
2	1956 Laolang (53°)	Sichuan Langjiu Group Co., Ltd.	1.059 ± 0.008	1.431
3	Jiabin Lang (50°)	Sichuan Langjiu Group Co., Ltd.	0.060 ± 0.002	0.081
4	Xiaohutuxian (52°)	Guizhou Xingyi Yunfeng Co., Ltd.	^a^ tr	- ^b^
5	Tianzhilan (42°)	Jiangsu Yanghe Distillery Co., Ltd	tr	- ^b^
6	Xiangquan (54°)	Jiugui Liquor Co., Ltd.	0.068 ± 0.001	0.092
7	Jinliufu Sixing (38°)	Wuliangye Group	0.064 ± 0.001	0.086
8	Jinliufu Hongsejingdian (38°)	Wuliangye Group	tr	- ^b^
9	Shuanggou Daqu (38°)	Shuanggou Distillery	0.091 ± 0.002	0.123
10	Niulanshan Bainian (38°)	Shunxin Agriculture Ture	tr	- ^b^
11	Jianzhuang Chenjiu (52°)	Wuliangye Group	tr	- ^b^
12	Laishigang Chenniang 3 Years (53°)	Laishigang Group	0.062 ± 0.001	0.084
13	Zhijiang Zhixin 5 Years (52°)	Zhijiang Group	0.081 ± 0.002	0.109
14	Jiannanchun (52°)	Sichuan Jiannanchun Jituan Co., Ltd.	tr	- ^b^
15	Liulingzui 3 (52°)	Liulingzui Group	0.100 ± 0.002	0.135
16	Zhonghua Dukang K3 (50°)	Luoyang Dukang Holdings Limited Official Website	0.052 ± 0.001	0.070
17	Yangshao Caitaofang Jiuliang Miaopin (52°)	Yangshao Co., Ltd.	0.553 ± 0.006	0.747
18	Mianrou Dukang (50°)	Luoyang Dukang Holdings Limited Official Website	0.065 ± 0.001	0.088
19	Shamochun Shengshi (42°)	Neimenggu Dahekou Co., Ltd.	0.064 ± 0.001	0.086
20	Luzhou LaojiaoTouqu (52°)	Luzhou Laojiao Co., Ltd.	tr	- ^b^
21	Luzhou Laojiao Chentouqu 8 Years (52°)	Luzhou Laojiao Co., Ltd.	tr	- ^b^
22	Hetao Laojiao Jinzun (42°)	Hetao Liquor	tr	- ^b^
23	Yingjia K6 (38°)	Yingjia Gongjiu Co., Ltd.	0.075 ± 0.003	0.101
24	Neimenggu Sorghum Blue Era (38°)	Neimenggu Dahekou Co., Ltd.	0.068 ± 0.001	0.092
25	Rouhe Shuanggou (42°)	Shuanggou Distillery	0.059 ± 0.001	0.080
26	Xinghuacun Baishun (45°)	Fenjiu Group	0.094 ± 0.001	0.127
27	Jingjiu Jixing (36°)	Wuliangye Group	0.065 ± 0.002	0.088
28	Guizhou Yuanjiang Zhenpin 9 Years (38°)	Guizhou Maotai Distillery Group Technology Development Company	0.068 ± 0.002	0.092
29	Guizhou DongcangYuanjiu 30 Years (38°)	Zhenpin Jiuye	0.302 ± 0.010	0.408
30	Xifeng Yucang (38°)	Xifeng Co., Ltd.	0.056 ± 0.001	0.076
31	Furuiwang (38°)	Furui Co., Ltd.	0.079 ± 0.001^c^	0.107
32	Laobaifen Fengtan 15 Years (38°)	Fenjiu Group	0.112 ± 0.001	0.151
33	Honghuaci Erguotou (56°)	Sanhe Fucheng Co., Ltd.	0.224 ± 0.042	0.303
34	Jingdu Yujiu (42°)	Shuangqinghe Co., Ltd.	tr	- ^b^
35	Zhoufuji Erguotou (42°)	Zhoufuji Co., Ltd.	tr	- ^b^
36	Mendaolv (62°)	Ningheyuan Co., Ltd.	tr	- ^b^
37	Weirenmin Fuwu (53°)	Guizhou Maotai Distillery Group	0.130 ± 0.002	0.176
38	Beijing Erguotou Qinghuaci (52°)	Jiuzhongjiu Co., Ltd.	0.064 ± 0.002	0.086
39	Zhougong Baisui (35°)	Huangjia Jingdu Co., Ltd.	0.052 ± 0.003	0.070
40	Tianshan Laobing (38°)	Tianshan Co., Ltd.	tr	- ^b^
41	Beijing Erguotou I (56°)	Tongquanyong Co., Ltd.	tr	- ^b^
42	Xianghe Ronghe Shaofang (53°)	Kweichow Moutai Co., Ltd.	1.712 ± 0.023	2.313
43	Dajinjiu (42°)	Dajin Co., Ltd.	tr	- ^b^
44	Jingdu Heitan (42°)	Huangjia Jingdu Co., Ltd.	0.066 ± 0.003	0.089
45	Wuliang Yuanjiu (50°)	Yuqiao Co., Ltd.	0.300 ± 0.054	0.405
46	Guocuijiu (52°)	Luzhou Guocui Co., Ltd.	0.052 ± 0.001	0.070
47	Hongdu Tezhen 25 Years (50°)	Hongdu Co., Ltd.	tr	- ^b^
48	Beijing Erguotou II (56°)	Shuangqinghe Co., Ltd.	0.075 ± 0.006	0.101
49	Beijing Fangzhuang Erguotou (52°)	Fangzhuang Co., Ltd.	tr	- ^b^
50	Laobeijing Erguotou (41°)	Duxing Co., Ltd.	tr	- ^b^
51	Sichuan Sorghumjiu I (40°)	Yaoquan Laojiao Co., Ltd.	tr	- ^b^
52	Guantoushan (40°)	Guantoushan Co., Ltd.	0.093 ± 0.001	0.126
53	Mengguwang (44°)	Mengguwang Co., Ltd.	0.090 ± 0.002	0.122
54	Caoyuan Andaqing (62.8°)	Andaqing Co., Ltd.	0.100 ± 0.004	0.135
55	Luchun (52°)	Luzhou Laojiao Co., Ltd.	tr	- ^b^
56	Yujingfang Shaojiu (38°)	Yujingfang Shaojiu Group	0.058 ± 0.002	0.078
57	Sichuan Sorghum II (42°)	Culiangfang Co., Ltd.	tr	- ^b^
58	Xianli Jianguo 60 Years (50°)	MaotaiJiucheng Co., Ltd.	tr	- ^b^
59	Longfeng (38°)	Longfeng Co., Ltd.	tr	- ^b^
60	Caoyuan Liema (62°)	Menggudao Co., Ltd.	tr	- ^b^
61	Shuijing Kongdong (52°)	Liuhuchun Co., Ltd.	0.066 ± 0.001	0.089
62	Gubei Chunliang (42°)	Gubeichun Co., Ltd.	0.050 ± 0.001	0.066
63	Banmasuo Chenniao 3 Years (65°)	Muniu Co., Ltd.	0.074 ± 0.001	0.100
64	Heitudi (38°)	Hecheng Co., Ltd.	tr	- ^b^
65	Hengshui Laobaigan (39°)	Hengshui Laobaigan Co., Ltd.	0.149 ± 0.005	0.201
66	Bancheng Laojiu (42°)	Qianlongzui Co., Ltd.	0.075 ± 0.001	0.101
67	Jingyanggang (38°)	Jingyanggang Co., Ltd.	1.900 ± 0.002	2.568
68	65667 Troops Tegong T99B (38°)	Beimao Co., Ltd.	0.250 ± 0.002	0.338
69	Huanghelong Laoliangfang (52°)	Huanghelong Group	0.100 ± 0.001	0.135
70	Guojiao 1573 (52°)	Luzhou Laojiao Co., Ltd.	tr	- ^b^
71	Mengzhilan M6 (40.8°)	Jiangsu Yanghe Distillery Co., Ltd	0.176 ± 0.006	0.238
72	Kouzijiao Zhencang 20 Years (41°)	Kouzi Yjiuye	0.052 ± 0.002	0.070
73	Moutai (53°) ^c^	Kweichow Moutai Co., Ltd.	0.851 ± 0.001 ^c^	1.150
74	Xifeng (55°) ^c^	Xifeng Co., Ltd.	0.818 ± 0.011 ^c^	1.105
75	Guojing 1# (65°) ^c^	Shandong Bandaojing Co., Ltd.	1.008 ± 0.018 ^c^	1.362
76	Gujing Yuanjiang (65°) ^c^	Anhui Gujing Group Co., Ltd.	2.237 ± 0.022 ^c^	3.023
77	Jinshiyuan Yuanjiang (59°) ^c^	Jiangsu King’s Luck Brewery Joint-Stock Co., Ltd.	0.932 ± 0.024 ^c^	1.259
78	Jinshiyuan (53°) ^c^	Jiangsu King’s Luck Brewery Joint-Stock Co., Ltd.	0.810 ± 0.017 ^c^	1.095
79	Jingzhi Guniang10 Years (52°) ^c^	Shandong Jingzhi Liquor Co., Ltd.	3.024 ± 0.025 ^c^	4.086
80	Jingzhi Guniang 5 Years (50°) ^c^	Shandong Jingzhi Liquor Co., Ltd.	3.286 ± 0.060 ^c^	4.440
81	Wuyue Duzun (52°) ^c^	Taishan Liuor Group Co., Ltd.	0.788 ± 0.006 ^c^	1.065
82	Jiuchao Chenxiang (42°) ^c^	Shandong Lanling Meijiu Co., Ltd.	0.910 ± 0.014 ^c^	1.230

^a^ tr: The concentrations of propyl lactate were between 0.025 mg∙L^−1^ and 0.050 mg∙L^−1^; ^b^ No OAVs because of the concentrations of propyl lactate was much less than LOQ; ^c^ the concentrations of propyl lactate were from the reported article [16].

As shown in Table 1, propyl lactate did occur in all Chinese Baijius under investigation, though the concentrations in some distillate drinks were between 0.050 mg∙L^−1^ (LOQ) and 0.025 mg∙L^−1^ (LOD). The top 5 distillate samples were Jingzhi Guniang 5 years (50°) (3.286 mg∙L^−1^), Jingzhi Guniang 10 years (52°) (3.024 mg∙L^−1^), Gujing Yuanjiang (65°) (2.237 mg∙L^−1^), “Jingyanggang” (38°) (1.900 mg∙L^−1^), and “Xianghe Ronghe Shaofang” (53°) (1.712 mg∙L^−1^).

### 2.3. Detection Threshold of Propyl Lactate and OAV Analysis

**Figure 3:**
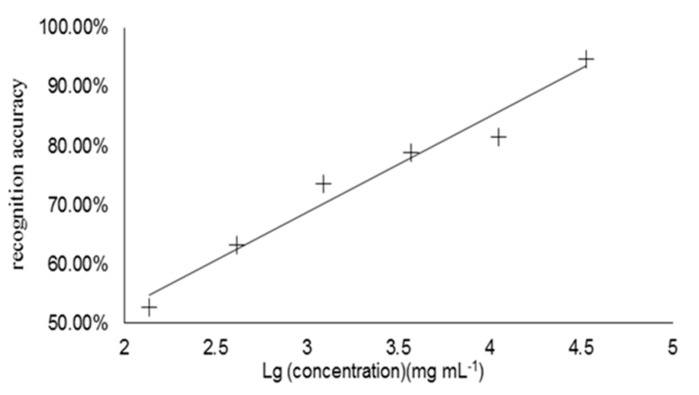
Scatter diagram by CF for determination of propyl lactate detection threshold. The X-axis represents the concentration of propyl lactate to the base 10 logarithm (X = LogA) and Y-axis is the correct recognition ratio, which is the ratio of the correct recognition numbers in total recognition numbers$\;\left(\text{Y}=\frac{\text{N}\left(\text{correct}\right)}{\text{N}\left(\text{totall}\right)}\right)$.

The determination of detection threshold of propyl lactate was conducted by the statistical analyses and the Curve Fitting, and the result was shown in Figure 3.

The calibration curve equation was *y* = 0.1623*x* + 0.6877, and the correlation coefficient R^2^ = 0.9663. The threshold was the corresponding X value (0.740 mg∙L^−1^) when Y = 66.67% [17].

### 2.4. Discussion

The unknown compound shown at 17.4 min in sample 33 was identified to be propyl lactate based on the comparison of mass spectra and retention time with the standard. Propyl lactate was also discovered in all other samples under investigation by the same method.

**Figure 4:**
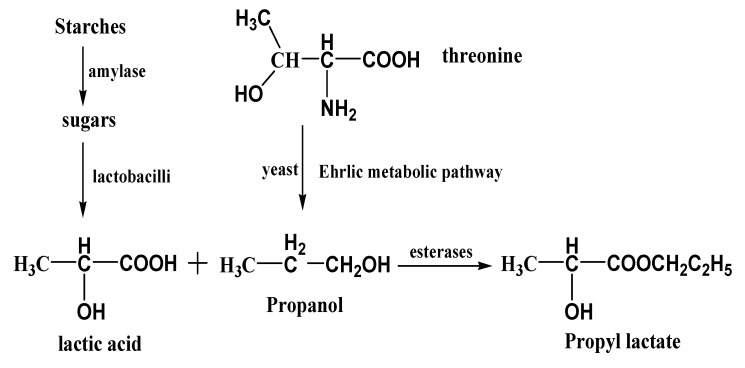
The pathway of propyl lactate formation by esterase catalyzation.

Propyl lactate was possibly formed through the esterification of lactic acid with propanol, both of which have been reported during the fermentation. There were a great number of lactobacilli during the fermentation process of Chinese spirit drinks, which could convert sugars to lactic acid [18,19]. Higher alcohols could be formed during the fermentation under aerobic condition from sugar or under anaerobic conditions from amino acids [20] since the raw materials, sorghum, rice, sticky rice, wheat and corn, were rich sources of amino acids. Propanol can be produced from threonine by yeast via the Ehrlich metabolic pathway [21]. Small amounts of propanol could also be formed by yeast through reduction of propanal. The esterification of lactic acid with propanol could be taken place directly or catalyzed by esterases during the fermentation and aging process of Chinese liquor production [20]. The esterases might be from yeasts, molds, or bacteria, which existed in “Daqu” or “Xiaoqu” [21]. Fan [22] also reported that the “Daqu” had high esterase activities. The main formation process of propyl lactate was as Figure 4.

The odor activity values (OAV) equaled to the ratio of the concentration of propyl lactate and its detection threshold value. If a compound has an OAV > 1.0, then it would contribute to the flavor of a product [23]. The OAVs of propyl lactate in 82 Chinese Baijius were listed in Table 1. There were 13 distillate samples with the OAVs of propyl lactate higher than 1, including 50° Jingzhi Guniang 5 years (4.440), 52° Jingzhi Guniang 10 years (3.024), 38° Jingyanggang (2.568), 53° Xianghe Ronghe Shaofang (2.313), and so on, whereas other 69 liquor samples with the OAVs lower than 1. Propyl lactate had a grape-like fruity, milk and ester odor [17]. The results indicated that propyl lactate was one of odor-active components of these 13 Chinese Baijius. Whether propyl lactate was a key odor compound or not based on more experiments and proofs, which we would be to study next.

## 3. Experimental Section

### 3.1. Chemicals

Chemicals and standards were GC grade with a high purity (>99.0%).The water was boiling for at least 0.5 h and redistilled twice before use. Methyl lactate (PubChem CID: 11040), used as internal standard (IS), and propyl lactate (PubChem CID: 92821) were obtained from Tokyo Chemical Industry CO., Ltd. (Shanghai, China). Absolute ethanol (PubChem CID: 702) was obtained from Merck (Darmstadt, Germany).

### 3.2. Spirit Drink Samples

A total of 72 spirit drinks, shown as Table 1, were obtained from different factories in China, or supermarkets, such as Wal-Mart and Carrefour in Beijing, China.

### 3.3. Qualitative and Quantitative Analysis by GC-MS

1.0 µL of Chinese spirit drinks was injected and analyzed by GC-MS with the full scan mode, and the occurrence of propyl lactate was confirmed by comparing its retention time and mass spectrum with the standards.

The concentrations of propyl lactate in these distillates were determined by the internal standard method with the selected ions monitoring mode of GC-MS. At first, a series of the standard solutions, such as 5.000 mg∙L^−1^, 2.500 mg∙L^−1^, 1.250 mg∙L^−1^, 0.625 mg∙L^−1^, 0.313 mg∙L^−1^, 0.156 mg∙L^−1^, 0.078 mg∙L^−1^, 0.039 mg∙L^−1^ and 0.020 mg∙L^−1^, were prepared with absolute ethanol and analyzed by GC-MS. Then 1.0 mL of each distillate drink sample with 10.0 µL of methyl lactate solution (100.000 mg∙L^−1^) were placed in 72 tightly closed sample vials, numbered 1 to 72, for GC-MS analysis. Finally, the concentrations of propyl lactate were calculated by the software of GC-MS. The conditions of GC-MS (6890A-5975C, Agilent technologies Co., Ltd., Beijing, China) were as follows.

GC conditions: DB-FFAP capillary column (30 m × 0.25 mm, 0.25 μm film thickness, Santa Clara, CA, USA); carrier gas, helium, 99.9995%; flow rate, 1.0 mL∙min^−1^; The oven temperature was programmed at 50 °C for 2 min, then raised to 100 °C at 6 °C∙min^−1^, then raised to 170 °C at 3 °C∙min^−1^ for 2 min, and then raised to 200 °C at 10 °C∙min^−1^ for 2 min, and finally raised to 230 °C at 15 °C∙min^−1^ for 5 min; inlet temperature, 250 °C; transfer line temperature, 250 °C; injection volume, 1 µL; split ratio, 20:1.

MS conditions: electron ionization source, 70 eV; ion source and quadruple temperatures, 230 °C and 150 °C, respectively; The monitored ions and other parameters of selected-ion-monitoring (SIM) mode were listed in Table 2.

**Table 2:** The monitored ions and other parameters.

Compound	Mode	Mass List or Range
Methyl lactate	Full Scan	50–500
	SIM	45, 75, 89,105
Propyl lactate	Full Scan	50–500
	SIM	45, 75, 117

### 3.4. Determination of the Detection Threshold of Propyl Lactate

3-AFC test was recommended as the national standard method for determination of Chinese Baijiu flavors thresholds by GB/T 22366-2008 [16], which is a general guidance for measuring odor, flavor and taste detection threshold, because of its higher efficiency and accuracy. Meanwhile, CF method was adopted as threshold calculation method, which was recommended by ASTM E1432-2004 Standard Practice. So 3-AFC and CF were selected to determine the detection threshold of propyl lactate in Chinese spirit drinks.

Most concentrations of alcohol in the distillate samples under investigation were 38 vol% or nearby, so 38% ethanol solution was used as the benchmark. A series of propyl lactate solutions were prepared for sensory evaluation with 38% ethanol solution, such as 0.137 mg∙L^−1^ (A_1_), 0.412 mg∙L^−1^ (A_2_), 1.235 mg∙L^−1^ (A_3_), 3.704 mg∙L^−1^ (A_4_), 11.111 mg∙L^−1^ (A_5_) and 33.333 mg∙L^−1^ (A_6_).

There were eighteen samples for sensory evaluation, which were equally divided into six groups. All these samples were placed in 10 mL tulip-like glass wine cups. Two samples of each group were the control samples and the remaining one had different concentration of propyl lactate in it. Each sample was marked in a random four-digit number. A group of 30 untrained and normal olfaction assessors were invited to determine propyl lactate detection threshold. All samples were assessed at room temperature. For each test, the assessors needed to pick out the one which was “very different from the references”, and wrote down the number. Each test was replicated 3 times, so that 90 responses were obtained for each testing concentration. Then the Curve Fitting (CF) was conducted by the statistical analyses, and the curve was drawn. When Y = 66.7%, the corresponding X value was the detection threshold value of propyl lactate.

## 4. Conclusions

In summary, this work reported the occurrence of propyl lactate in 72 Chinese Baijius for the first time. The concentration of propyl lactate ranged from 0.050 to 1.900 mg∙L^−1^ in 72 Chinese Baijius. The detection threshold of propyl lactate in 38% ethanol solution was 0.740 mg∙L^−1^. Based on OAV analysis in this research, propyl lactate had much contribution to the aroma of 13 Chinese Baijius , including 50° Jingzhi Guniang 5 years (4.440), 52° Jingzhi Guniang 10 years (3.024), Jingyanggang (2.568), Xianghe Ronghe Shaofang (2.313), and 1956 Laolang (1.431).
